# From Brain Organoids to Networking Assembloids: Implications for Neuroendocrinology and Stress Medicine

**DOI:** 10.3389/fphys.2021.621970

**Published:** 2021-06-10

**Authors:** Evanthia A. Makrygianni, George P. Chrousos

**Affiliations:** ^1^University Research Institute of Maternal and Child Health and Precision Medicine, National and Kapodistrian University of Athens, Athens, Greece; ^2^Center for Adolescent Medicine and UNESCO Chair on Adolescent Health Care, First Department of Pediatrics, School of Medicine, National and Kapodistrian University of Athens, Aghia Sophia Children’s Hospital, Athens, Greece

**Keywords:** brain organoids, assembloids, neuroendocrinology, stress response system, stress-related disorders, hypothalamus, locus caeruleus

## Abstract

Brain organoids are three-dimensional cultures that contain multiple types of cells and cytoarchitectures, and resemble fetal human brain structurally and functionally. These organoids are being used increasingly to model brain development and disorders, however, they only partially recapitulate such processes, because of several limitations, including inability to mimic the distinct cortical layers, lack of functional neuronal circuitry as well as non-neural cells and gyrification, and increased cellular stress. Efforts to create improved brain organoid culture systems have led to region-specific organoids, vascularized organoids, glia-containing organoids, assembloids, sliced organoids and polarized organoids. Assembloids are fused region-specific organoids, which attempt to recapitulate inter-regional and inter-cellular interactions as well as neural circuitry development by combining multiple brain regions and/or cell lineages. As a result, assembloids can be used to model subtle functional aberrations that reflect complex neurodevelopmental, neuropsychiatric and neurodegenerative disorders. Mammalian organisms possess a highly complex neuroendocrine system, the stress system, whose main task is the preservation of systemic homeostasis, when the latter is threatened by adverse forces, the stressors. The main central parts of the stress system are the paraventricular nucleus of the hypothalamus and the locus caeruleus/norepinephrine-autonomic nervous system nuclei in the brainstem; these centers innervate each other and interact reciprocally as well as with various other CNS structures. Chronic dysregulation of the stress system has been implicated in major pathologies, the so-called chronic non-communicable diseases, including neuropsychiatric, neurodegenerative, cardiometabolic and autoimmune disorders, which lead to significant population morbidity and mortality. We speculate that brain organoids and/or assembloids could be used to model the development, regulation and dysregulation of the stress system and to better understand stress-related disorders. Novel brain organoid technologies, combined with high-throughput single-cell omics and gene editing, could, thus, have major implications for precision medicine.

## Introduction

Scientists have been seeking to understand mechanisms of human disease since the time of Hippocrates. However, the traditional use of animal models has been somewhat problematic, because of evolutionary divergence. On the other hand, immortalized human cell lines (e.g., HeLa cells) are characterized by chromosomal instability and restricted tissue specificity (Adey et al., 2013). Stem cell-derived *in vitro* life model systems have been the focus of recent efforts. Stem cells are defined as cells that have the ability to divide indefinitely and produce different cellular types as their progeny (Tajbakhsh, 2009; Zakrzewski et al., 2019). Human embryonic (hESCs) and induced pluripotent (hiPSCs) stem cells, collectively called human pluripotent stem cells (hPSCs), can be induced to spontaneously undergo differentiation and morphogenesis, mimicking the formation of embryonic tissues. This process can be achieved by aggregating in 3D structures, called embryoid bodies (EBs). Within EBs, morphogenesis can be directed toward specific germ layers, when specific growth factors are applied (Lancaster and Huch, 2019).

Organoids are spatially organized 3D tissues that consist of multiple cell types which self-organize through similar processes as these observed *in vivo* (i.e., cell-sorting and spatially restricted lineage commitment) and, thus, their progressive organization is highly reminiscent of the actual organ morphogenesis (Lancaster and Knoblich, 2014). Brain organoids are hPSC-derived organoids which are representative of tissue architecture (i.e., they contain progenitor, neuronal and glial cells) and developmental trajectory of the fetal human brain (Qian et al., 2019). These organoids are of great interest, as the living human brain is technically and ethically inaccessible for *in vivo* studies (Benito-Kwiecinski and Lancaster, 2019).

This review will focus on brain organoids and their potential use in studying and understanding the stress system, a highly conserved neuroendocrine system, which is essential for systemic homeostasis. We first introduce briefly the history of brain organoid generation, then we discuss recent advances in brain organoid technologies and, finally, we speculate on their potential applications as model systems of neurodevelopment and neurological or psychiatric disease. In this context, we introduce neuroendocrinology and the neuroendocrine stress system and stress-related disorders and discuss the potential applications of brain organoid technologies in these fields.

## CNS Embryology

**FIGURE 1:**
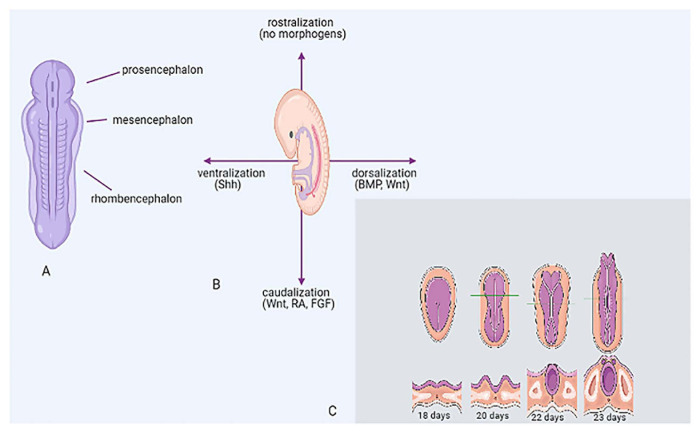
CNS embryology. **(A)** Brain vesicles giving rise to prosencephalon, mesencephalon, rhombencephalon (axial view). **(B)** Establishment of axes through morphogen gradient, *in vivo* (lateral view). **(C)** Embryogenesis of the neural tube around a fluid-filled lumen and development of brain vesicles at 18, 20, 22, and 23 days. BMP, bone morphogenetic protein; FGF, fibroblast growth factor; RA, retinoic acid; SHH, sonic hedgehog.

The central nervous system (CNS) originates from the neural ectoderm, which gives rise to the neural plate, which further differentiates into the neural tube. The latter is organized around a fluid-filled lumen, representative of the brain ventricles (Figure 1C). Morphogens are secreted by multiple organizing centers and their gradient defines the axes, i.e., the ventral-dorsal axis is influenced by Sonic Hedgehog (SHH)-Wnt-bone morphogenetic proteins (BMPs), whereas the rostral-caudal axis is defined by retinoic acid (RA) and fibroblast growth factors (FGFs) (Figure 1B). Initially, the neural tube is divided into prosencephalon (forebrain), mesencephalon (midbrain), and rhombencephalon (hindbrain) (Figure 1A). As embryogenesis proceeds, the prosencephalon further differentiates into telencephalic and diencephalic structures, while the rhombencephalon differentiates to form metencephalon and myelencephalon, which give rise to the pons, the cerebellum, the medulla oblongata and the spinal cord. Neurons are generated from neural stem cells (NSCs) which are situated next to ventricular walls. During neurogenesis, NSCs give rise to neural progenitors as well as more differentiated neural cells, such as intermediate progenitors and neurons. According to differentiation stage, neural cells migrate further outwards, and thus multilayered, stratified structures are formed. These structures have different number of layers, dependent on CNS topology and specialization e.g., the medulla, the cerebral cortex and the optic tectum (superior colliculus) have three, six and seven layers, respectively (Stiles and Jernigan, 2010; Lancaster and Knoblich, 2014; Clevers, 2016; Agirman et al., 2017). Neural induction to rostral identities (forebrain) represents the default pathway of differentiation and is achieved *in vivo* via inhibition of BMP/Nodal signaling (Suzuki and Vanderhaeghen, 2015).

## From PSCs to Brain Organoids

The brain has an intrinsic self-organizing capacity, i.e., if neuroepithelial cells are derived from PSCs, they will subsequently self-organize spontaneously into laminar structures. Zhang et al. (2001) discovered that hESCs could form neural rosettes (2D neural tube-like structures), revealing the self-organization potential of neural progenitors. Neural rosettes recapitulated apical-basal polarity and exhibited spontaneous radial organization, but they could not mimic the overall organization of the developing brain due to their 2D nature (Lancaster and Knoblich, 2014). Subsequently, efforts were made to recapitulate brain tissue organization with 3D cultures. Eiraku et al. (2008) used a 3D aggregation culture (serum-free culture of embryoid body-like aggregates with quick reaggregation, SFEBq) to generate ESC-derived, self-organized cortical tissues with apicobasal polarity. Kadoshima et al. (2013) further improved this method, obtaining telencephalic structures with multiple laminar and separated cortical zones as observed in the embryonic cortex during the second trimester. Initial cultures used dual SMAD and Wnt inhibition for neural induction as well as for direct differentiation toward a telencephalic fate (Lancaster and Huch, 2019). Lancaster et al. (2013) showed that a broader brain regional identity could be generated by simply providing an extracellular matrix (ECM, Matrigel) to EBs without any signaling molecules. The resultant cerebral organoids (heterogeneous neural organoids) contained several different brain regions within individual organoids (Lancaster et al., 2013). After that, several studies generated region-specific organoids by using patterning factors. These organoids included cortical spheroids (Pasca et al., 2015), hippocampal (Sakaguchi et al., 2015), hypothalamic and pituitary (Suga et al., 2011; Ozone et al., 2016), cerebellar (Muguruma et al., 2015), and midbrain organoids (Jo et al., 2016). Subsequently, the assembly of region-specific (ventral and dorsal forebrain) organoids showed that ventral forebrain organoid interneurons have the ability to migrate to dorsal forebrain, resembling *in vivo* brain development (Bagley et al., 2017; Birey et al., 2017; Xiang et al., 2017).

## Brain Organoid Methodologies

Methods for generating brain organoids can be classified into unguided and guided. Unguided methods are based on spontaneous morphogenesis, intrinsic differentiation capacities and developmental programs within hPSCs; hPSC-derived EBs are grown in ECM and self-organize into distinct brain regions via endogenous signaling (Qian et al., 2019). The resultant *cerebral organoids* contain heterogeneous tissues, resembling brain regions (Lancaster and Knoblich, 2014). During this self-organizing process, extensive neuroepithelial conformations may arise with a wide range of cell lineage identities such as forebrain, midbrain, hindbrain, choroid plexus and retina (Lancaster et al., 2013; Quadrato et al., 2017). Interestingly, neuroepithelial tissue within cerebral organoids was capable of developing signaling centers and local tissue patterning (Benito-Kwiecinski and Lancaster, 2019). Nevertheless, the described spontaneous differentiation is characterized by stochasticity. As a result, each lineage and cell type can be represented by unpredictable proportions and a heterogeneous arrangement across organoid batches as well as across hPSC lines. Similarly, proportion and spatial organization of various interacting brain regions can be heterogeneous and unpredictable (Qian et al., 2019). On the other hand, during guided methods, excessive regional heterogeneity is overcome and regional identity is limited to a specific brain region. By using external patterning factors at an early stage (which may be removed at later stages), hPSCs are induced to differentiate toward desired lineages, mimicking *in vivo* development. Different *region-specific organoids* can be fused into *assembloids*. However, the addition of excessive patterning signals, during guided approaches, may reduce organoid complexity and mask important aspects of development and subtle phenotypes. The choice between unguided and guided methodologies will depend on the specific focus of research and weighing between diversity and consistency. Unguided organoids may facilitate the study of cellular diversity during brain development. Among guided methods, brain region-specific organoids may be suitable for the exploration of less heterogeneous brain cytoarchitectures, whereas assembloids may allow the study of molecular and functional interactions and the cross-talk between specific brain regions (Benito-Kwiecinski and Lancaster, 2019; Qian et al., 2019).

## Brain Assembloids

**FIGURE 2:**
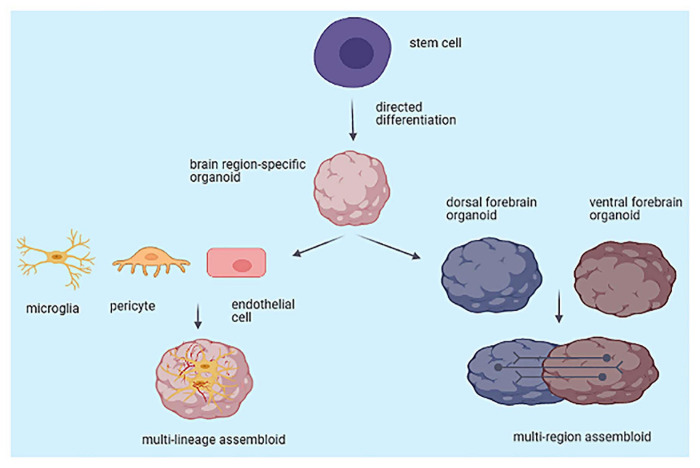
Generation of multi-region and multi-lineage brain assembloids from stem cells.

Assembloids are the next generation of brain organoids that can combine multiple brain regions and/or cell lineages in 3D culture. They can be used to model interactions between different brain regions, to capture cell–cell interactions, and to study the assembly of neural circuits. To model interactions between cortical glutamatergic neurons and GABAergic interneurons, several groups developed separate organoids resembling the dorsal and ventral forebrain and then fused them together into a *multi-region assembloid* (Bagley et al., 2017; Birey et al., 2017; Xiang et al., 2017; Sloan et al., 2018) (Figure 2). Similarly, Xiang et al. (2019) generated thalamocortical assembloids that recapitulate thalamic development and may model cortico-thalamic interactions, which shape cortical circuits. During brain development, extensive reciprocal projections between the thalamus and the cortex are formed and are involved in sensory-motor processing, attention and arousal, as the thalamus is an information relay hub. Thalamic dysfunction has been implicated in neurodevelopmental disorders, including autism, schizophrenia and epilepsy (Xiang et al., 2019). Assembloids can also be used to assemble other region-specific organoids, such as the cortex to the striatum or midbrain, e.g., to study corticostriatal interactions, as there is evidence that several neurodevelopmental, neuropsychiatric and movement disorders might be attributed to disordered corticostriatal connectivity (Shepherd, 2013). Nevertheless, further work is needed to reliably assess connectivity *in vitro* and to learn to what extent assembloids can capture more subtle inter-regional changes, associated with the so-called connectopathies. Besides input from other brain regions, neural development and function is shaped by interactions with other cell types, including microglia, astrocytes, oligodendrocytes, or mesoderm-derived blood vessels. It is increasingly recognized that neuroimmune and neurovascular interactions are important for brain development. Neural/non-neural interactions can be modeled *in vitro* by adding non-neural cells in brain region–specific organoids, at various stages of differentiation, to form *multilineage assembloids* (Paşca, 2019; Qian et al., 2019) (Figure 2).

## Cellular Interactions Within Brain Organoids

### Neuronal Interactions

The cerebral cortex comprises neurons (glutamatergic pyramidal neurons and GABAergic interneurons) as well as glial cells (astrocytes, oligodendrocytes and microglia). The maturing forebrain can be subdivided into dorsal forebrain (glutamatergic neurons) and ventral forebrain (GABAergic interneurons); the former gives rise to the cortex, while the latter comprises the various divisions of the ganglionic eminence (GE), from which cortical interneurons derive. Most interneurons originate from medial and caudal GE, in which they acquire specific identities (e.g., parvalbumin, somatostatin, and calbindin). Then, they start a migratory journey to populate the developing dorsal forebrain, via tangential migration. This process starts in humans around gestational age 15 weeks and continues throughout the second year of life (Sloan et al., 2018). After having undergone activity-dependent maturation, interneurons are then integrated into neural circuits, characterized by an excitation/inhibition balance, which plays a central role in the development of the CNS. Aberrations in this fine balance may contribute to neurodevelopmental/neuropsychiatric disorders (Sloan et al., 2018; Marton and Pasca, 2019). Studies in forebrain organoids have reproducibly demonstrated that dorsal organoid glutamatergic neurons form synapses with the migrating ventral organoid interneurons and integrate into a microcircuit with increased morphological complexity, in a similar way as in the cerebral cortex (Pasca et al., 2015; Birey et al., 2017; Yoon et al., 2019). Interestingly, the 3D nature of forebrain assembloids is essential for migration (Birey et al., 2017). Additionally, organoid neurons have been shown to interact with host neurons and develop functional connectivity after transplantation into adult mouse brain (neurological chimeras). This implies that host neurons may influence the function, maturation and differentiation of the transplanted neurons (Daviaud et al., 2018; Mansour et al., 2018).

### Astrocyte Interactions

It has been increasingly recognized that glial cells are important for neuronal development. Astrocytes play a central role in brain homeostasis and synaptic plasticity, and have been characterized as the housekeeper of the CNS (Logan et al., 2019). They affect neurons pleiotropically through extensive interactions, which influence neuronal development, maturation and survival as well as formation of synapses (Marton and Pasca, 2019). The exact nature of this influence (i.e., direct intercellular or indirect via secreted molecules) is unknown. Abnormal astrocyte function has been mechanistically implicated in the etiology of neuropsychiatric disorders (Sloan et al., 2018). Temporally, astrogenesis takes place after neurogenesis in *in vitro* 3D cultures, and astrocytes increase in number and become more mature over time as observed *in vivo*. Long-term organoid cultures allow for advanced astrocytic maturation, simulating *in vivo* developmental stages; this maturation would be difficult to obtain in a 2D culture (Sloan et al., 2017). Currently, mature organoid astrocytes can only be studied by isolation and placing into tissue culture plates. Exploring how astrocytes behave within organoids (e.g., by imaging methods) would elucidate the mechanisms through which astrocytes modulate neuronal development (Marton and Pasca, 2019).

### Oligodendrocyte Interactions

Initially, brain organoids lacked oligodendrocytes. Although several attempts have been made to produce oligodendrocytes from hiPSCs in 2D cultures, various functional aspects of oligodendrocytes – including myelination – are difficult to study in 2D (Marton and Pasca, 2019). Alternatively, oligodendrocytes have been generated in addition to astrocytes and neurons in organoids; this allows the formation of compact myelin through interactions with neuronal processes. These cultures can be used to model white matter formation and its disorders as well as interactions between oligodendrocytes and other types of cells (Madhavan et al., 2018; Marton et al., 2019).

### CNS and Non-CNS Lineage Cell Interactions in Brain Organoids

#### Microglial Interactions

Microglia are the main immune cells of the CNS. Microglial precursors arise from the yolk sac, migrate to the brain and quickly diverge from macrophages, which reside in other tissues; this process takes place under the influence of unknown brain-derived signals (Li and Barres, 2018). The presence of microglia in organoids would allow the study of the roles of microglia in phagocytosis of damaged cells, release of cytokines as well as synaptogenesis and elimination of synapses (Marton and Pasca, 2019). Because it is difficult to reproduce the development of microglia in culture, attempts to study microglial-neural interactions have converged into two methods. According to the first one, microglial cells are produced from hiPSCs, outside organoids, and then are integrated into the latter to form multi-lineage assembloids; this allows the study of morphological and functional aspects of microglia in 3D, including response to injury (Abud et al., 2017; Paşca, 2019; Song et al., 2019a). In the second method, minimally patterned organoids – which contain multiple germ layer progenitor cells – are used to derive microglial cells (Ormel et al., 2018). Microglia generation *in vitro* is essential for modeling neuroimmunological disorders, including multiple sclerosis (MS) and autoimmune encephalitis (Marton and Pasca, 2019). Assembloids that contain microglia and neurons could be valuable for the study of immune-mediated pathways during synaptic elimination. Additionally, mutations in genes that are expressed in myeloid cells, including monocytes and microglia, have been associated with neuropsychiatric diseases, but the mechanism is unknown (Paşca, 2019).

### Vascular Cell Interactions/Brain Organoids With a Functional Vascular-Like System and BBB Characteristics

The wall of brain blood vessels consists of the mesoderm-derived endothelial cells (ECs), whereas pericytes are also found in capillaries (Alberts et al., 2002; Marton and Pasca, 2019). The presence of blood vessels within brain organoids is essential for reducing the levels of cellular stress and death and for generating mature organoids. Additionally, the blood–brain barrier (BBB) as well the influence of neural activity on blood flow could be studied in brain organoids that contain blood vessels (Marton and Pasca, 2019). Several attempts have been made to generate blood vessels within organoids. Mansour et al. (2018) used a mouse host to transplant human-derived brain organoids; organoids were invaded with host blood vessels. Pham et al. (2018) vascularized brain organoids with patient-derived ECs. Song et al. (2019b) fused cortical spheroids and isogenic endothelial spheroids from hiPSCs alongside mesenchymal stem cells (MSCs). Cakir et al. (2019) generated human cortical organoids with ETS variant transcription factor 2 (ETV2) induction. In accordance with their hypothesis that ETV2 expression induces ECs and structures similar to blood vessels in organoids, they engineered hESCs to ectopically express ETV2. The resultant vascularized organoids formed functional vasculature, when implanted in mice (Cakir et al., 2019). Bergmann et al. (2018) created BBB organoids by co-culturing primary brain ECs, pericytes and astrocytes. The main components of BBB are brain microvascular endothelial cells (BMECs), pericytes, neurons and astrocytes. Unique features of the BBB, including the high transendothelial electrical resistance (TEER), have been attributed to tight junctions formed by BMECs (Marton and Pasca, 2019). The production of BMECs *in vitro* can be challenging. Hence, further research is needed to generate these cells *in vitro* (Lu et al., 2021).

## Brain Organoids and Cellular Stress

By using single-cell transcriptomics, Bhaduri et al. (2020) concluded that cortical organoids contain a smaller number of cell subtypes, compared to the human cortex and this may be attributed to ectopic activation of cellular stress pathways, which leads to disordered cell-type specification. Immature, broader cell classes, not representative of cellular subtypes can be contained within brain organoids. Consequently, brain organoids may not be reliable models for the study of developmental processes, disease phenotypes that depend on specific cell types, and inter-cellular connectivity. Of note, cellular stress and impaired cellular specificity are reduced when organoids are transplanted into mouse cortex (Bhaduri et al., 2020).

## Sliced Cortical Organoids

Because cortical organoids lack functional blood vessels, organoid cells show impaired viability, due to inadequate supply of oxygen and nutrients via surface diffusion; this prevents organoids from reaching the cytoarchitecture of late developmental stages. Qian et al. (2020) used a slicing method to develop a sliced neocortical organoid model with well-separated upper and deep cortical layers. This model prevented cell death within organoids and allowed growth over long-term culture, thus recapitulating late stage human cortical developmental features. This method may be used to improve cell viability in 3D culture systems and may also be applied to other brain regions or other organs. Interestingly, slicing could be combined with hyperoxia, which has already been employed in organoid protocols. This combination would further reduce cellular hypoxia and improve cell viability, while distinct cortical layers would be achieved at the same time (Qian et al., 2020).

## Polarized Brain Organoids

Brain organoids lack topographical organization along dorsoventral and anteroposterior axes. During CNS development, combinations of morphogens (e.g., SHH, Wnt, and BMPs) are secreted by organizing centers, in complex spatiotemporal patterns; this allows cells to acquire discrete regional identities as a function of their position. This process could be mimicked in brain organoids/assembloids by precise positioning of signaling centers via engineering methods (Cederquist et al., 2019; Miura and Pasca, 2019). Cederquist et al. (2019) generated a signaling center within forebrain organoids by assembling them with a group of SHH-secreting cells. Organoids became polarized into several forebrain regions. Further studies are needed to test whether differentiating organizer-like cells from hPSCs and then combining them with brain organoids could be used as a general approach to establish topographies across all regions of the CNS. Polarized organoids provide the opportunity to study a wide range of phenotypes in a single organoid system. They could be used to model complex neurodevelopmental disorders, in which altered regional specification during forebrain patterning has been hypothesized or to study the effects of hypothalamic peptidergic system on the cerebral cortex (Cederquist et al., 2019; Miura and Pasca, 2019).

## Cortical Organoids for Modeling the Development of Cerebral Cortex and Their Limitations

The human cortex is profoundly different from those of other species and is the most uniquely evolutionary expanded region of the human brain. Hence, hPSC-derived cortical organoids are advantageous, compared to animal models. Nevertheless, these organoids are far from identical to *in vivo* and *in situ* cerebral cortex. Firstly, conventional cortical organoids are much smaller than human cerebral cortex. Their size can reach up to ∼5–6 mm in diameter, whereas the human cortex is about 15 cm in diameter, with gray matter being 2–4 mm thick (Qian et al., 2019) and cortical surface area being around 2,000 cm^2^ (Hofman, 2014). Secondly, they lack vascularization and contain a necrotic core, which further limits their viable thickness. Thirdly, although cortical organoids recapitulate the organization of neural progenitor zones in a spatiotemporal manner (i.e., deep layer neurons are formed first, followed by upper layer neurons), there is extensive mixing and co-localization; this leads to restricted neuronal spatial layering and incomplete cortical lamination (Benito-Kwiecinski and Lancaster, 2019; Qian et al., 2019).

Distinct cortical layers are established around the second trimester of pregnancy and thus conventional organoids are suitable models of this embryonic period; this is congruent with the finding that organoids have similar transcriptomic and epigenetic profiles to those of early human fetal cortex up to the second trimester (Camp et al., 2015; Luo et al., 2016; Qian et al., 2016; Quadrato et al., 2017). Recent advances in organoid technologies have led to organoids with well-separated layers, which is a feature of human cortical development at late stages (Qian et al., 2020).

Organoid neurons are able to form synapses, with a synaptic density similar to that observed in fetal brain. These neurons show spontaneous and coordinated firing activity, resulting in neuronal networks with self-organized firing patterns (Quadrato et al., 2017; Benito-Kwiecinski and Lancaster, 2019). Accordingly, forebrain assembloids generate functional, interacting, excitatory and inhibitory neurons and provide the opportunity to study cell migration, neuronal circuit formation and interregional interactions, which shape brain development. However, these networks are less mature compared to adult neuronal networks (Sloan et al., 2018). Interestingly, assembloids show improved firing frequency compared to non-fused organoids, indicating that fusion confers additional neuronal properties, not obtainable in a single organoid (Seto and Eiraku, 2019). Nevertheless, further studies are needed to examine the genesis and regulatory mechanisms of functional circuits in the fusion system (Xiang et al., 2019).

Brain organoids lack sensory input (Amin and Paşca, 2018; Velasco et al., 2020). During early embryonic development, there are patterns of spontaneous neuronal activity that synchronize local and large-scale cortical networks; these later guide the establishment of global thalamocortical and intracortical networks. The earliest neuronal networks are autonomous and transient. After sensory input from the periphery has reached the cortex, circuits are reshaped and matured by integration of sensory input with spontaneous neuronal activity (Molnár et al., 2020).

Brain organoids lack gyrification, possibly because they do not have the ability to reach the developmental stage where this takes place. Alternatively, this may be due to the small size of brain organoids, as cortical folding is associated with the surface area and the thickness of CP (cortical plate) (Qian et al., 2019). Synthetic biomaterial-based methods have been used to model the physics of the folding brain (Karzbrun et al., 2018) and control the geometrical and biomechanical properties of brain organoids (Oksdath et al., 2018). Additionally, it has been increasingly recognized that the biochemical composition of the ECM may influence the biomechanical properties of the brain and may have a central role in the cellular differentiation and brain architecture. Matrigel, currently used as ECM in brain 3D culture technologies, partly reflects the complex composition of brain ECM. New biosynthetic matrices may provide control over the physicochemical and mechanical characteristics of the microenvironment of brain organoids (Oksdath et al., 2018).

One of the most significant limitations of brain organoids is organoid-to-organoid and batch-to-batch variability, especially when unguided methods are used (Velasco et al., 2020). Recent efforts have focused on generating reproducible brain organoids (Velasco et al., 2019; Yoon et al., 2019). By using scRNA-seq (single cell RNA sequencing), Velasco et al. (2019) characterized cells of different mature cortical organoids at 3 and 6 months. Cortical organoids derived from different stem cells yielded similar ratios of different cellular types, and were highly reproducible across cell lines and batches. Similarly, Yoon et al. (2019) generated highly reliable and replicable cortical spheroids (region-specific organoids), regardless of initial cell line and experiment. However, generating reproducible organoids of other brain regions remains challenging. This may be explained by the fact that the default differentiation state of neural progenitors is to become cortex and this can be fulfilled even outside the embryonic brain (Velasco et al., 2020).

## Disease Modeling

Due to their versatility, brain organoids are suitable for modeling diseases of either genetic or environmental etiology (Adams et al., 2019). They have been extensively used to model structural neurodevelopmental brain disorders, attributed to disordered progenitor cell migration, including microcephaly, macrocephaly and lissencephaly (Lancaster et al., 2013; Gabriel and Gopalakrishnan, 2017; Li et al., 2017a, b). However, due to their inability to form cortical folds, modeling diseases such as Miller–Dieker syndrome (characterized by prominent lissencephaly) is difficult (Bershteyn et al., 2017; Iefremova et al., 2017). Additionally, effects of neurotrophic pathogens on brain development can be modeled. Interestingly, when brain organoids are exposed to Zika virus, neural progenitor cells (NPCs) are preferentially infected, leading to cell death and reduced organoid size (Cugola et al., 2016; Dang et al., 2016; Garcez et al., 2016; Qian et al., 2016).

Modeling neurodevelopmental disorders which are characterized by less prominent or no structural malformations is more difficult. However, the use of organoids, in this case, can provide insights into disease-related cellular and molecular mechanisms. For example, an imbalance between excitatory/inhibitory neurons (associated with overexpression of FOXG1) has been shown in forebrain organoids from autism spectrum disorder (ASD)-derived PSCs (Mariani et al., 2015; Choi et al., 2017). On the other hand, in ihPSCs-derived dorsal-ventral forebrain assembloids from individuals with Timothy syndrome (a genetic, multisystem disorder characterized by ASD features), defects in interneuron migration have been observed (Birey et al., 2017). Brain organoids have also been generated to model Rett syndrome (Mellios et al., 2018), tuberous sclerosis (TS) (Blair et al., 2018) and schizophrenia (Stachowiak et al., 2017; Kathuria et al., 2020). The modeling of diseases associated with abnormalities in network-level activity among distant brain regions remains a challenge (Seto and Eiraku, 2019).

Brain organoids have been utilized to model neurodegenerative diseases. However, the majority of neurodegenerative diseases present later in life, are age-related, and are usually progressive. Hence, brain organoids may not be accurate neurodegeneration models. Additionally, neurovascular interactions are indispensable for the modeling of neurodegenerative disorders, as they recapitulate the neurodegenerative microenvironment (Paşca, 2019; Grenier et al., 2020). Several groups have generated cortical organoids to model Alzheimer disease (AD) and recapitulated the molecular phenomena that are observed in AD, such as aggregation of β-amyloid, hyperphosphorylation of tau protein, and endosomal abnormalities (Lee et al., 2016; Raja et al., 2016; Lin et al., 2018; Gonzalez et al., 2018).

Midbrain organoids containing functional dopaminergic neurons have been generated, mainly for modeling Parkinson’s disease (PD). These organoids, especially when disease-specific mutations are introduced or pharmacological treatment is applied to induce neurodegeneration, may be used as disease models. Midbrain organoids could also be valuable in the context of cell-replacement therapies (Kim et al., 2019; Smits et al., 2019; Kwak et al., 2020). Additionally, brain organoids capable of modeling both early development and features of neurodegeneration could be revolutionary in the study of chronic neuropsychiatric disorders, which are often characterized by alterations in both neurodevelopmental and neurodegenerative processes (Grenier et al., 2020). Moreover, hypothalamic organoids containing nuclei-like clusters of neuropeptidergic neurons could be promising models for studying metabolic disorders and obesity (Qian et al., 2016; Qian et al., 2018; Rajamani et al., 2018).

### Brain Organoids and Aging

Brain organoids are derived from either ESCs or iPSCs. Apart from aging-induced somatic mutations, many features of aging including epigenetic changes, DNA/oxidative damage and reduced telomere length are reverted during iPSC reprogramming (Cornacchia and Studer, 2017). However, there is evidence that several epigenetic characteristics of aging are conserved after reprogramming of iPSCs that are derived from patients with syndromes associated with premature aging (Agarwal et al., 2010; Batista et al., 2011; Andrade et al., 2012; Grenier et al., 2020). As a result, accelerated aging could be studied in brain organoids that are derived from these iPSCs. Additionally, aging could be induced by introducing disease (including neurodegeneration and progeria)-associated mutations into PSCs via genome editing. Alternatively, it has been proposed that aging could be induced by exposing cells to aging-associated stress such as ROS (reactive oxygen species), pro-inflammatory molecules and radiation (Hu et al., 2018; Grenier et al., 2020). However, it remains unclear which of these factors would optimally induce cellular aging-related changes (Hu et al., 2018). Interestingly, age-related changes of neurons are retained after direct neuronal reprogramming (i.e., the direct conversion of cells from one lineage to another without going through the pluripotent stage) and, thus, induced neuron models may allow the modeling of age-associated diseases (Mertens et al., 2015; Mertens et al., 2018).

Of note, 3D cultures allow long culture periods (e.g., 60 weeks), which can be used to study chronological aging *in vitro* (Grenier et al., 2020). Additionally, there is increasing evidence that there may be an interaction between ECM and senescent cells (Levi et al., 2020). Age-related changes in the components of the ECM may disrupt cellular homeostasis and cellular aging may change the composition of ECM. As a result, organoids are more suitable to model *in vitro* the microenvironment of aging, compared to cell lines (Birch, 2018; Hu et al., 2018).

In summary, although brain organoids provide a window into understanding complex neurological diseases, at this time, they are simplistic and at an early stage, while they may be biased models because of their *in vitro* nature. Further improvements, including generation of organoids with well-defined connectivity between several brain regions, acceleration of functional maturation, efficient incorporation of other cell and tissue types (e.g., glia and vasculature), and effective modeling of aging could lead to more comprehensive and realistic models. Supplementary Table 1 shows advances in brain organoid methodologies to date.

## Single-Cell Omics and Brain Organoids

Sequencing of bulk tissues has been increasingly replaced by single cell genomics, which is moving rapidly from scRNA-seq to single-cell multi-omics. This is because scRNA-seq snapshots may provide insight into cellular diversity, but cannot explain why and how a cell adopts a certain state (Packer and Trapnell, 2018; Schier, 2020).

On the other hand, combined multi-modal analysis of genome, transcriptome, epigenome, chromatin organization and proteome as well as information about spatial localization of cells can shed light on multiple aspects of cellular identity (Burgess, 2019; Efremova and Teichmann, 2020). Genome sequencing combined with scRNA-seq may elucidate genotype-phenotype correlations and the phenotypic impact of genetic variants. Because other genes and molecules can affect gene transcription, scRNA-seq may provide evidence about potential gene-regulatory networks. Joint transcriptomics and chromatin accessibility analysis could reveal novel cell states and investigate the activity of TFs and enhancer elements (Efremova and Teichmann, 2020). Additionally, by using single cell proteomics, ligands, receptors as well as downstream signaling molecules and lineage-specific TFs could be quantified and inter- and intra-cellular signaling network maps could be constructed. Simultaneous measurement of proteins and RNAs could associate cell-signaling with gene expression; poor association between gene and protein expression is indicative of post-transcriptional modifications (Efremova and Teichmann, 2020).

Organoids consist of various cellular types and states and are characterized by high organoid to organoid variability. Applying single-cell omics to brain organoids could improve our mechanistic understanding of human brain development and disease-related phenomena, through the study of lineage relationships and regulatory networks (Camp and Treutlein, 2017; Atamian et al., 2021).

## Gene Editing in Brain Organoids

Brain organoids can be genetically modified either transiently or permanently. Stable modifications (by lentivirus, transposon and CRISPR/Cas9 systems) are typically performed at early developmental stages (hPSCs, or EBs), whereas transient modifications [by adeno-associated virus (AAV) and electroporation-based techniques] are performed at later stages (mature organoids) (Fischer et al., 2019).

Among stable methods, older nuclease-based methods have been replaced by CRISPR/Cas9. Currently, the major application of CRISPR-Cas9 is to model monogenic neurodevelopmental and neurodegenerative diseases as well as oncogenesis, by either introducing pathogenic mutations into control hiPSCs, or utilizing patient hiPSCs to generate isogenic controls (Fischer et al., 2019). However, the modeling of complex disorders remains a challenge. In the future, a combination of transient and stable gene editing methods may allow modeling complex diseases and developmental processes, as transient methods can be more versatile and efficient in mature brain organoids (Camp and Treutlein, 2017; Fischer et al., 2019).

Another future application of genetically modified brain organoids could be cell-lineage tracing to explore the developmental history of individual cells. This could be accomplished by single-cell sequencing and labeling via lineage-specific expression of fluorescent proteins (Fischer et al., 2019). Additionally, introduction of DNA mutations with CRISPR/Cas9 could create genetic scars, which could be used as cell-specific markers (Camp and Treutlein, 2017).

## Neuroendocrinology

**FIGURE 3:**
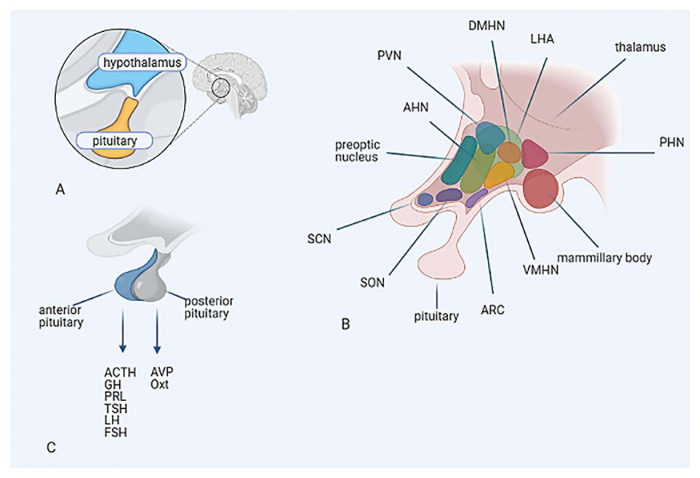
**(A)** Anatomical characterization of the hypothalamus and the pituitary. **(B)** Structural organization of the hypothalamic nuclei. **(C)** Hypothalamic control of hormonal output of the pituitary. Hypothalamic nuclei: AHN, anterior hypothalamic nucleus; ARC, arcuate nucleus; DMHN, dorsomedial hypothalamic nucleus; LHA, lateral hypothalamic area; PHN, posterior hypothalamic nucleus; PVN, paraventricular nucleus; SCN, suprachiasmatic nucleus; SON, supraoptic nucleus; VMHN, ventromedial hypothalamic nucleus. Pituitary hormones: ACTH, adrenocorticotropic hormone; AVP, arginine vasopressin; FSH, follicle-stimulating hormone; GH, growth hormone; LH, luteinizing hormone; Oxt, oxytocin; PRL, prolactin; TSH, thyroid-stimulating hormone.

The hypothalamus is highly conserved anatomically and functionally throughout vertebrates, due to its essential role in regulating homeostasis and behavior. It regulates pituitary gland secretion (Figures 3A,C), but also regulates sleep, body temperature, feeding and aging through connections via the autonomic nervous system (ANS) and other pathways. Pituitary hormones and their targets regulate multiple functions, including fluid balance, stress response, reproduction, growth, metabolism and pain. The hypothalamus and the hypophysis are developmentally and functionally connected (Rizzoti et al., 2016; Xie and Dorsky, 2017) (Figure 3A).

The morphogenesis of the hypothalamus is complex, compared to other regions of the brain, because its structural organization lacks clear landmarks (Bedont et al., 2015; Rizzoti et al., 2016). In contrast to the columnar organization of other brain regions, the hypothalamus consists of various nuclei, organized in a 3D network (Figure 3B).

Secretion of gradients of morphogens is essential for patterning of the hypothalamus during development. Wnt, SHH, BMP, FGF, Nodal and Notch signaling determine the hypothalamic identity. During this process, variable gradients of multiple TFs are secreted to fine-tune the patterning and define the various hypothalamic nuclei (Bedont et al., 2015; Xie and Dorsky, 2017).

The posterior lobe of the pituitary arises from the neuroectoderm, whereas the anterior pituitary derives from non-neural ectoderm. Pituitary morphogenesis is regulated by interactions between the presumable posterior lobe, the infundibulum (an early diencephalic structure) and the Rathke’s pouch (derived from oral ectoderm). Additionally, the anterior pituitary placode and the hypothalamic anlage interact with each other; SHH, Wnt, BMP, and FGF signaling seem to determine this interaction and the hypophyseal patterning (Takuma et al., 1998; Brinkmeier et al., 2007; Zhu et al., 2007).

Rathke’s pouch expresses the TFs LHX3 and LHX4 (Rizzoti and Lovell-Badge, 2005). Additionally, TFs including TBX19, POU1F1, GATA2 are essential for subsequent lineage commitment and differentiation to adrenocorticotropin (ACTH)-, growth hormone (GH)-, prolactin (PRL)-, thyroid-stimulating hormone (TSH)-, luteinizing hormone (LH)- and follicle-stimulating hormone (FSH)-producing cells (Rizzoti and Lovell-Badge, 2005; Kelberman et al., 2009; Suga, 2019).

The neuroendocrine system comprises many functionally diverse cell types. Mature endocrine cells can be difficult to obtain, thus, the *in vitro* study of specific subtypes of human neuroendocrine cells has not been feasible; this could be overcome by the use of hPSCs. There have been several efforts to generate neuroendocrine tissues and organoids from PSCs (Suga et al., 2011; Ozone et al., 2016; Ogawa et al., 2018; Kasai et al., 2020). Ogawa et al. (2018) induced arginine vasopressin (AVP)-secreting neurons from hESCs. Suga et al. (2011) and Ozone et al. (2016) recapitulated pituitary development in 3D. Kasai et al. (2020) generated a functional hypothalamus-pituitary unit. The culture methods used in the above studies have been shown to recapitulate effectively the hypothalamic and hypophyseal embryogenesis, and therefore could have applications in developmental neuroendocrinology. Adhya et al. (2018) suggested that brain organoids could be used to study the impact of neurosteroids on brain development. This study could lead to a better understanding of developmental phenomena, such as the sexual dimorphism of the brain as well as the relation of neurosteroids to neurodevelopmental and neurodegenerative diseases (Adhya et al., 2018).

Another potential use of hPSC-derived hypothalamic-pituitary cells could be in regenerative medicine, although, at this time, generated tissues are not as functional as normal tissues (Kasai et al., 2020). PSC-derived ACTH-producing cells have been shown to function efficiently, if they are extrinsically controlled by releasing factors or small molecules, even after ectopic transplantation. However, in ectopic transplantation, hypothalamic corticotropin-releasing hormone (CRH) release does not affect the grafts. Orthotopic transplantation of hormone-producing cells could be advantageous in the future (Rizzoti et al., 2016; Suga, 2019).

## Pituitary Organoids

Several studies have reported methods to differentiate hPSCs into anterior pituitary, *in vitro*. Suga et al. (2011) produced functional 3D pituitary tissues from mouse embryonic cells (mESCs). After that, Ozone et al. (2016) produced regulator hormone-responsive pituitary tissue from hESCs. In addition to corticotrophs, they generated somatotrophs with appropriate GH secretion in response to growth hormone-releasing hormone (GHRH) and somatostatin, which had not been achieved by Suga et al. (2011) (Supplementary Table 1).

## Hypothalamic-Pituitary Units

Kasai et al. (2020) generated a functional hypothalamic-pituitary unit from 3D-cultured hiPSCs. During embryonic development, the hypothalamus interacts with the anterior pituitary and this interaction is considered to be essential for pituitary development (Takuma et al., 1998; Scully and Rosenfeld, 2002; Rizzoti and Lovell-Badge, 2005; Zhu et al., 2007). In that context, Kasai et al. (2020) juxtaposed anterior pituitary and hypothalamic neurons and observed that their proximity increased the ACTH secretion capacity of the pituitary, but also resulted in regulation of ACTH by hypothalamic CRH. The authors concluded that the hypothalamus plays a crucial role in the development and maturation of the anterior pituitary. *In vivo*, anterior pituitary secretion is regulated by the hypothalamus, depending on the micro-environment, so that homeostasis is maintained; ACTH cells are stimulated by hypothalamic CRH, whereas they are inhibited by glucocorticoids. The aggregates generated by Kasai et al. (2020) responded appropriately to both CRH and glucocorticoids. The results of this study suggest that ACTH^+^ cells function under the control of CRH^+^ cells in this 3D, *in vitro* model (Kasai et al., 2020).

## The Neuroendocrine Stress Response System

Stress is a state of disrupted organismal homeostasis, due to physical or emotional forces, called stressors. When these forces exceed a certain threshold, adaptive compensatory physiologic responses are activated, which constitute the neuroendocrine stress or adaptation response (Chrousos, 2007, 2009). This response is essential for survival and is remarkably consistent, so that it has been postulated that a discrete, dedicated neuroendocrine system has evolved for its coordination (Chrousos and Gold, 1992). The stress response allows an organism to make the necessary physiological and metabolic changes that are needed to cope with homeostatic demands (Miller and O’Callaghan, 2002).

The stress response system has two major arms: (a) the CRH system and (b) the locus caeruleus^∗^-norepinephrine (LC-NE) system. The major central components of these arms are the *paraventricular nucleus (PVN)* of the hypothalamus and the pontine *locus caeruleus (LC)* alongside their projections to the *brainstem autonomic nuclei*. The peripheral components are the pituitary-adrenal axis and sympathetic nervous (SNS)/sympathoadrenal system as well as components of the parasympathetic nervous system (PSNS).

**FIGURE 4:**
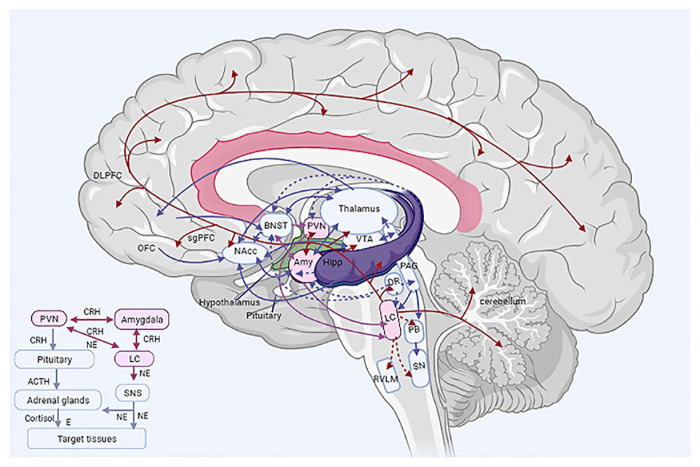
The stress system. Main figure: central components of the stress system. Inset (bottom left corner): Simplified overview of the stress system (central and peripheral). ACTH, adrenocorticotropic-releasing hormone; Amy, amygdala; BNST, bed nucleus of the stria terminalis; CRH, corticotropin-releasing hormone; DLPFC, dorsolateral prefrontal cortex; DR, dorsal raphe nucleus; E, epinephrine; Hipp, hippocampus; LC, locus caeruleus; NE, norepinephrine; NAcc, nucleus accumbens; OFC, orbitofrontal cortex; PAG, periaqueductal gray matter; PB, parabrachial nucleus; PFC, prefrontal cortex; PVN, paraventricular nucleus of the hypothalamus; RVLM, rostral ventrolateral medulla; sgPFC, subgenual prefrontal cortex; SN, solitary nucleus; SNS, sympathetic nervous system; VTA, ventral tegmental area. Solid line arrows represent excitatory projections, whereas dashed line arrows represent inhibitory ones. Double-headed arrows represent reciprocal connections. Efferent LC pathways are depicted in red.

The CRH system is widespread throughout the brain (hypothalamic and extra-hypothalamic CRH system), but it is best characterized in the PVN and amygdala (central nucleus of the amygdala, CeA) (Chrousos and Gold, 1992; Chrousos, 2007; Gold et al., 2015b). The amygdala CRH system induces anxiety, but also activates the hypothalamic CRH system and the LC-NE system. The hypothalamic CRH system regulates the HPA axis and activates the LC-NE system (Gold et al., 2015b) (Figure 4, Inset).

The PVN plays a central role as an initiator of endocrine and autonomic responses, which are essential for maintaining homeostasis and adapting to stressors. It has a magnocellular, parvocellular and an autonomic subdivision. The first two control the hormonal output of the posterior and anterior pituitary, respectively (Figure 3C). Neurons of the autonomic subdivision project to the brainstem autonomic nuclei and the spinal cord. The PVN receives both excitatory and inhibitory input, mediated by glutamate and GABA, respectively. Limbic inputs from the prefrontal cortex (PFC) and amygdala, involved in the stress response, are relayed to the PVN, primarily via the bed nucleus of the stria terminalis (BNST) and the dorsomedial hypothalamic nucleus (DMH). Interestingly, direct projections from the CeA to the PVN are limited. Stress causes rapid activation of neural pathways afferent to the PVN, resulting in rapid CRH and AVP release to the pituitary portal circulation. Induction of AVP expression enhances stress-induced activation of HPA axis and the effect of CRH on ACTH (Chrousos and Gold, 1992; Benarroch, 2005).

Activation of the CRH system promotes arousal, fear-related behaviors and inhibits sleep, feeding and reproductive activity, which could be distractive during threatening conditions. Additionally, during the stress response, activation of CRH neurons leads to activation of pro-opiomelanocortin (POMC) neurons of the hypothalamic arcuate nucleus, which inhibit both the CRH neurons and the LC-NE system and mediate opioid receptor-induced analgesia (Chrousos and Gold, 1992; Chrousos, 2007).

The LC is the principal noradrenergic nucleus of the CNS, regulating arousal and autonomic activity by extensive projections to numerous structures of the CNS and peripheral nervous system (PNS). These projections can be either excitatory, through activation of α_1_-adrenoreceptors, or inhibitory via activation of α_2_-adrenoreceptors (Samuels and Szabadi, 2008b). The LC projects to the entire cortex to increase cortical activity, and is reciprocally connected with the PFC, regulating cognition, attention and vigilance. Also, it is reciprocally connected with the amygdala to process the emotional valence of the stimuli and promote fear/anxiety as well as increase sympathetic activity in the presence of threatening stimuli. It is reciprocally connected to the serotoninergic dorsal raphe nucleus (DR) and also projects to the thalamus to further promote wakefulness. It receives input from the midbrain periaqueductal gray (PAG), which is involved in sleep-wakefulness and REM sleep regulation. It projects to the pendunculopontine (PPT) and laterodorsal tegmental nuclei (LDT) of the brainstem to regulate wakefulness and inhibit REM sleep. Additionally, it inhibits the GABAergic neurons of the hypothalamic ventrolateral preoptic area (VLPO) to maintain arousal, thus simultaneously disinhibiting the hypothalamic histaminergic projections to the cortex. It inhibits the hypocretin (HRCT) neurons of lateral hypothalamus to suppress feeding. It projects to the hippocampus, which is mostly inhibitory on both the amygdala and the PVN CRH system (Chrousos, 2007; Samuels and Szabadi, 2008a, b). Also, it projects to the cerebellum to enhance motor performance and planning. It inhibits the preganglionic parasympathetic nuclei and the rostral ventrolateral medulla (RVLM) to control cardiovascular function and projects to sympathetic and parasympathetic neurons of the spinal cord (Samuels and Szabadi, 2008a, b).

Additionally, the LC is connected to the *mesocortical* and *mesolimbic dopaminergic pathways*, which are involved in motivation, reward and drug addiction. The former connects the midbrain ventral tegmental area (VTA) to the PFC and the latter connects the VTA to the ventral striatum in the basal ganglia (including the nucleus accumbens, NAcc) (Chrousos and Gold, 1992; Chrousos, 2007). Afferent excitatory fibers from the VTA project to the LC via the mesocaeruleal pathway which contributes to the maintenance of arousal, whereas efferent fibers project from the LC to both the VTA and the NAcc. The VTA has reciprocal connections with the PFC (Samuels and Szabadi, 2008a, b; Haber, 2011; Ferrucci et al., 2013; Gold, 2015a). The NAcc seems to be the main input nucleus of the basal ganglia, and receives input from the amygdala, hippocampus, thalamus and PFC, participating in a cortico-pallido-thalamo-cortical loop (Salgado and Kaplitt, 2015).

The LC-NE system has many similar effects to those of the CRH system, e.g., arousal, inhibition of neuro-vegetative functions, loss of affective and cognitive flexibility and reciprocally activates the amygdala and the CRH system (Gold et al., 2015b). The CRH and the LC-NE systems seem to be part of a positive feedback loop, where activation of one system tends to also activate the other. These interactions could be explained by projections from the PVN CRH neurons to the LC-NE system and vice versa by noradrenergic projections of the LC-NE system to the PVN (Figure 4, Inset). Furthermore, the CRH and the LC-NE systems seem to respond in a similar way to several neurochemical modulators, i.e., both serotonin and acetylcholine (Ach) are excitatory to CRH neurons and the LC-NE system, whereas GABA, opioids and glucocorticoids are inhibitory to both systems (Chrousos and Gold, 1992; Chrousos, 2007).

The PFC participates in the stress system. The function of sgPFC (subgenual PFC) – a term used interchangeably with subgenual anterior cingulate cortex (sgACC) (Drevets et al., 2008; Price and Drevets, 2012) – is moderately diminished during normal stress to disinhibit the CRH and the LC-NE systems and consequently to promote anxiety and arousal, while diminishing appetite and sleep. Additionally, the dorsolateral prefrontal cortex (DLPFC) and the orbitofrontal cortex (OFC) are moderately inhibited, thus decreasing cognitive regulation of anxiety and information processing concerning reward/pleasure (Gold, 2015a; Gold et al., 2015b).

The BNST, located in the basal forebrain, is the center of a network that connects the amygdala and the PVN. It is important for the regulation of the HPA response to stress, and is considered to be a component of the “extended amygdala”, but also a node which connects stress-related loci with the reward system. The BNST is interconnected with the amygdala, DR, hippocampus, hypothalamus, NAcc, VTA, thalamus, PFC. It also receives input from brainstem noradrenergic neurons, by which the BNST exerts inhibition on the HPA response to stress (Crestani et al., 2013; Stamatakis et al., 2014; Lebow and Chen, 2016; Goode and Maren, 2017). The HPA axis may be activated when inhibitory BNST neurons – which project to the PVN – are disinhibited. Responsiveness to stress stimuli depends on the duration of exposure (acute versus chronic stress activates different cells).

The PAG is a complex structure that coordinates the antinociceptive, behavioral and autonomic reactions to stress and injury. It connects the forebrain to the brainstem by receiving input from the cortex, the amygdala and hypothalamus and projects to the brainstem LC and autonomic nuclei. Stimulation of the dorsolateral PAG by escapable stress causes hypertension, tachycardia and aggressive behavior consistent with the fight-or-flight response, whereas stimulation of the ventrolateral PAG by non-escapable stress causes the opposite response, including bradycardia, hypotension and passive behavior. The PAG is reciprocally connected with the CeA and also projects to both the NAcc and the VTA as well as the thalamus (Benarroch, 2012).

The DR and the median raphe nucleus (MR) contain the majority of the forebrain–projecting serotoninergic neurons. The DR has reciprocal projections to anxiety-related structures including the CeA, BNST, PAG, mPFC (medial prefrontal cortex), and also receives input from the DMH and PVN. Serotoninergic activity depends on a fine balance between excitatory and inhibitory inputs to serotoninergic neurons, which is constantly changing (Hornung, 2003; Hale et al., 2012; Pollak Dorocic et al., 2014).

The paraventricular nucleus of the thalamus (Pa) seems to be a component of the stress system. There is evidence that the Pa is connected to the amygdala, BNST, NAcc, and sgPFC. Interestingly, hypothalamic HRCT projections to the Pa, via the amygdala and BNST, may facilitate HPA axis response to novel stress (after repeated and chronic stress) and regulate the facilitation/habituation balance. Because the Pa has a few direct projections to the PVN, regulation of the HPA axis by the Pa may be accomplished through the BNST to the PVN (Hsu et al., 2014).

New CNS loci are implicated increasingly in the stress response; for instance, the lateral habenula, an anti-reward diencephalic structure, seems to be involved in the stress system. The lateral habenula has been found to be connected reciprocally with the PVN, but the precise nature of these interactions remains unknown. This structure also interacts with dopaminergic and serotoninergic neurotransmission (Gold and Kadriu, 2019).

Figure 4 only partially depicts the complexity of the stress system.

## Disorders of the Stress System

The stress system is characterized by circadian rhythmicity and also responds to stressors on demand. During brief, controllable stress, the stress response is characterized by several short-term, temporarily beneficial, adaptive mechanisms, which activate this response only when it is needed (Chrousos, 2007, 2009). On the other hand, chronic, uncontrollable stress might affect development, growth and metabolism and may have a detrimental impact on many physiological systems, leading to neurobehavioral, metabolic, cardiovascular or autoimmune disorders. Genetic and epigenetic factors that determine the susceptibility or resilience of individuals to stress, environmental factors as well as exposure during critical developmental periods, but also the intensity and duration of stress may influence the development and severity of stress-related disorders (Chrousos, 2007, 2009). Stress induces altered neurogenesis and structural remodeling of the brain, including replacement of neurons and remodeling of dendrites and synapses (McEwen, 2007). Additionally, the stress itself causes transient or permanent epigenetic changes, which may alter gene expression and may determine resilience or susceptibility to stress (e.g., genes regulating the HPA axis, including genes for CRH, AVP, and the glucocorticoid receptor) (Nestler, 2012, 2014; Gold, 2015a).

## Depression as a Model of Dysregulation of the Stress Response

The stress response and major depression (MD) share major brain circuitries and mediators, including the PFC, amygdala, the LC-NE and CRH system, which participate in multiple inter-relating feedback loops. Hence, many of the features of MD reflect dysregulation of the stress response (Gold and Chrousos, 2002; Gold, 2015a). In melancholic depression, hypothalamic CRH hypersecretion leads to hypercortisolism and activation of the LC-NE system, whereas the gonadal, GH and thyroid axes are inhibited. Activation of the amygdala CRH system promotes anxiety and fear and activates the PVN CRH and the LC-NE systems. CRH activates the secretion of NE and vice versa, due to the interconnection of the CRH and LC-NE systems (Chrousos, 2007; Gold, 2015a). Inhibition of the sgPFC leads to disinhibition of the amygdala and vice versa (Gold et al., 2015b). The NAcc is suppressed by profound hypercortisolemia, leading to inability to adaptively alter reward-seeking behavior, to anhedonia and loss of motivation. On the other hand, mild cortisol elevation during normal stress increases the activity of the NAcc. Dopaminergic and serotonin neurotransmission are reduced. Premature aging and death may be attributed to a general dyshomeostatic state, which is characterized by activation of the CRH system, increased sympathetic activity, increased secretion of pro-inflammatory cytokines in both the brain and the periphery, insulin resistance and a prothrombotic state (Gold and Chrousos, 2002; Gold, 2015a; Gold et al., 2015b). Furthermore, chronic, uncontrollable stress and depression are both characterized by decreased neuroplasticity and neurogenesis, in which brain-derived neurotrophic factor (BDNF) signaling is disordered (Autry and Monteggia, 2012).

## Disorders of the LC-NE System

Stress alters LC neurons, and changes are dependent on its intensity and duration. A mild stress of short duration causes axonal sprouting, whereas prolonged, severe stress leads to axonal retraction or degeneration, possibly attributed to increased secretion of glucocorticoids (Nakamura and Sakaguchi, 1990; Nakamura et al., 1991).

Dysfunction of the LC-NE system is strongly correlated with several neurodegenerative disorders, including AD and PD, in which LC noradrenergic neurons undergo selective and early degeneration. Similar pathological changes are described in both AD and PD, indicating that AD and PD may be the two poles of a spectrum of neurodegenerative disorders, associated with LC-NE neuronal loss (Samuels and Szabadi, 2008b). Because the LC-NE system regulates attention, arousal and mood, degeneration of the LC may account for several neurobehavioral symptoms observed in both AD and PD, such as anxiety, depression and sleep disorders (Weinshenker, 2018). Additionally, LC-NE neurons have neuromodulatory and neuroprotective effects on the dopaminergic neurons of the substantia nigra and, thus, when the LC is compromised, it further contributes to neurodegeneration and the development of PD. Of note, patients with a history of depression show more profound neuronal loss within the LC (Samuels and Szabadi, 2008b).

Interestingly, the cognitive symptoms of schizophrenia may be attributed to the interaction between genetic susceptibility and dysfunction of the LC-NE system, which is triggered by stress (Mäki-Marttunen et al., 2020). Besides NE, disrupted neuromodulator signaling may contribute to alterations in LC-NE system activity in AD and PD, but also in chronic stress (Weinshenker, 2018).

## Specification of Noradrenergic Neurons

The generation and specification of noradrenergic neurons in the CNS and PNS, though poorly characterized, seems to be mediated by very similar transcriptional control mechanisms. In particular, the bHLH gene TFs Mash1 and Phox2b have been shown to be essential and sufficient for the generation of the LC and sympathetic ganglia. Previous studies have demonstrated *in vivo* that BMPs are essential for sympathetic neuron development and that the TFs Mash1, Phox2a, Phox2b, and dHAND are downstream regulators of BMPs in the sympathetic lineage.

In LC noradrenergic neurons, expression of Phox2a precedes and is essential for the later induction of Phox2b, which is followed by expression of dopamine beta-hydroxylase (DBH) and tyrosine hydroxylase (TH). BMP5 and BMP7 are expressed in the dorsal neuroepithelium, in proximity to Phox2-expressing cells, and they have been identified as likely candidates in LC generation. FGF8 is also essential for specification of noradrenergic neuron progenitors, early in neural tube development (Vogel-Höpker and Rohrer, 2002).

## Generation of Noradrenergic Neurons *In Vitro*

Mong et al. (2014) generated noradrenergic neurons from mESCs by forced expression of Phox2b, under the signaling influence of FGF8 and BMP7. Nevertheless, when they repeated their experiment in hESCs, there was lower expression of noradrenergic markers; BMP7 did not promote noradrenergic marker expression in hESCs. However, forced expression of Phox2b in hESCs, which were cultured with FGF8, promoted generation of noradrenergic cells (Mong et al., 2014). Kirino et al. (2018) generated sympathetic neurons from hPSCs. *In vivo*, sympathetic neurons originate from trunk neural crest cells (NCCs) cells, which in turn derive from neuromesodermal progenitor cells (NMPs). The authors concluded that BMPs and RA are essential for induction of Phox2b-expressing NCCs (Kirino et al., 2018). Eura et al. (2020) produced brainstem organoids that contained NCCs, midbrain and hindbrain progenitors as well as noradrenergic, dopaminergic and cholinergic neurons.

## Challenges and Opportunities: Using Brain Organoids to Model the Stress System and Stress-Related Disorders, Toward a Unified Theory of Stress

The stress response is orchestrated by a sophisticated system that consists of various interacting CNS structures, groups of neurons and circuits, which extend from the neocortex to primitive structures of the limbic system and participate in multiple regulatory loops (Chrousos and Gold, 1992; Gold and Chrousos, 2002; Chrousos, 2007, 2009; Gold, 2015a) (Figure 4). Among them, the hypothalamus is remarkably anatomically and functionally conserved among vertebrates (Xie and Dorsky, 2017). The LC is the major noradrenergic nucleus with numerous projections to almost all brain regions (Samuels and Szabadi, 2008a, b; Schwarz and Luo, 2015). Dysregulation of the stress system and the associated epigenetic changes may account for numerous complex diseases.

Despite the complexity of the stress system, brain organoids could be invaluable in the modeling of the stress response and stress-related disorders. Advances in brain organoid technologies include functional hypothalamic-pituitary organoids (Kasai et al., 2020), human brainstem organoids containing noradrenergic neurons (Eura et al., 2020), fused forebrain and thalamocortical assembloids (Birey et al., 2017; Bagley et al., 2017; Xiang et al., 2017, 2019), midbrain organoids (Jo et al., 2016; Monzel et al., 2017; Kim et al., 2019; Kwak et al., 2020), oligocortical spheroids with oligodendrocytes (Madhavan et al., 2018), hippocampal organoids (Sakaguchi et al., 2015), assembloids integrated with isogenic microglia (Song et al., 2019a), vascularized organoids with BBB properties (Cakir et al., 2019), polarized forebrain organoids (Cederquist et al., 2019), sliced forebrain organoids (Qian et al., 2020) (Supplementary Table 1). Central noradrenergic neurons and peripheral sympathetic neurons have been generated from hESCs (Mong et al., 2014; Kirino et al., 2018). On the other hand, LC-NE system or amygdala organoids have not been generated to date.

Modeling inter-cellular and inter-regional interactions within the stress system would necessitate the co-culture/assembly of multiple components of the stress system into *multi-region assembloids*. More subtle interactions and the developmental spatial topography of specific regions could be recapitulated by *polarized organoids/assembloids*. Initially, fused assembloids of the PVN, the LC, amygdala and PFC could be generated. However, the optimal goal would be the assembly of functional *whole brain polarized assembloids*, which would enable the elucidation of new stress-associated CNS loci. Additionally, non-neural cells, e.g., microglia to study neural/non-neural cellular interactions, and vascular cells to provide vascularization (which is essential for mature organoids) would be needed (*multi-region/multilineage assembloid*s).

However, it should be kept in mind that current organoid technologies are not able to accurately recapitulate *in vivo* functional connectivity between various distant brain regions. Furthermore, comprehensive understanding of the programs that drive the differentiation of various cells and regional morphogenesis in the CNS is lacking. Hence, identifying the minimal signals that are necessary for specification and self-organization would be essential for the development of other region-specific organoids (e.g., LC-NE system and amygdala organoids) as well as *polarized multi-region brain structures in vitro*.

Interestingly, the assembly of organoids from different tissue types could recapitulate the interaction between the brain and other organs toward *multi-organ assembloids* (e.g., hypothalamic-pituitary-adrenal or LC-NE system/adrenal assembloids). Of note, no hPSC-derived adrenal organoids have been generated to date. Poli et al. (2019) developed an *in vitro* human fetal cell model, representative of the adrenal gland components.

The generation of “*stress system organoids*” could provide a window of opportunity for basic and translational research. What are the molecular mechanisms that delineate the molecular stress response within individual region-specific organoids? How are these molecular signals integrated among the various region-specific organoids within the “*stress assembloids*” to establish a physiological stress response? How do the interactions of neural and non-neural cells within region-specific organoids, but also among different region-specific organoids within the “*stress system assembloids*”, delineate the development of the stress system and how does the early environment (prenatal, perinatal, and postnatal) affect this process? What are the genetic marks that confer vulnerability or resilience to stress? What are the associated epigenetic changes? Are they inherited? Are there differences between acute and chronic, mild and severe stress? How are the various components of the stress system engaged differently in different contexts?

The combination of brain organoids with multi-modal single-cell omics and lineage tracing could provide information about different cell types, their developmental trajectories and cell lineage as well as gene regulatory and signaling networks and could identify new cell types (Camp and Treutlein, 2017; Efremova and Teichmann, 2020). Insertion of fluorescent tags and reporter systems in PSCs (before the generation of organoids) could be used to study inter-cellular connectivity and cellular migration (Camp and Treutlein, 2017; Fischer et al., 2019).

Profiles of control *vs*. disease organoids could be compared. Initially, a representative stress-related disorder, such as depression, could be chosen to compare with controls. After that, other candidate disease groups could be studied to define whether dysregulation of the stress system is mechanistically implicated in the specific phenotype. Lineage-coupled single cell omics combined with CRISPR/Cas9 mutagenesis could make possible the localization of network perturbations, determine dysregulated genes and elucidate mechanisms of cell communication, regulation of cell specification as well as how environmental factors affect these processes during development (Camp and Treutlein, 2017; Fischer et al., 2019). Computational methods, including multi-modal deep learning and network-based fusion could further clarify causal relations between different omics layers and could be used to study genotype-phenotype correlations and to associate transcriptional with epigenetic phenomena that determine cellular phenotypes (Efremova and Teichmann, 2020; Schier, 2020).

We speculate that dysregulation of the stress system is involved in the pathophysiology of numerous complex systemic disorders, including neurodegeneration. We also speculate that the stress system is a ubiquitous, interspersed, conserved system which affects pleiotropically multiple CNS and peripheral targets, as a major regulator of systemic homeostasis and as a protagonist in dyshomeostasis (cacostasis), in the context of a unified theory of stress. The integration of the above methods into a model of the stress system would have a profound impact on precision medicine, in the near future.

## Conclusion

Despite recent advances in brain organoid technologies, existing cultures are far from perfect; thus, there is still a need for organoids that accurately reflect the characteristics of the human brain regarding regional and cellular diversity, connectivity, myelination, polarization, vascularization and, generally, reproducibility. Additionally, more mature organoids mimicking later developmental stages as well as the aging brain are needed. The generation of 3D, *in vitro* models of the stress system could have a considerable impact on the mechanistic explanation of the pathophysiology of a great number of stress-related disorders, which cause significant morbidity and mortality, as well as on developmental neuroendocrinology and beyond.

^∗^Locus caeruleus means the blue spot/place in Latin. *Caeruleus* means *blue* in classical Latin^1^. It probably derives from the classical Latin word *caelum* which means *sky*^2^. Locus caeruleus is dictated in the list of Latin expressions and English equivalents in Terminologia Anatomica^3^, which is the current edition of Nomina Anatomica^4^.

## Author Contributions

EM conceived and designed the work, collected data, analyzed and interpreted data, drafted the article, critically revised the article, and finally approved of the version to be published. GC conceived and designed the work, collected data, analyzed and interpreted data, critically revised the article, and finally approved of the version to be published. Both authors contributed to the article and approved the submitted version.

## Conflict of Interest

The authors declare that the research was conducted in the absence of any commercial or financial relationships that could be construed as a potential conflict of interest.

## Footnotes

1 1http://www.perseus.tufts.edu/hopper/resolveform?type=exact&lookup=caeruleus&lang=la

2 https://en.wiktionary.org/wiki/caeruleus

3 http://terminologia-anatomica.org/en/Search?query=locus%20caeruleus

4 https://en.wikipedia.org/wiki/Locus_coeruleus#Etymology

## Abbreviations

ACTH: adrenocorticotropin
AD: Alzheimer disease
ANS: autonomic nervous system
ASD: autism spectrum disorder
AVP: arginine vasopressin
BBB: blood–brain barrier
BMECs: brain microvascular endothelial cells
BMP: bone morphogenetic protein
BNST: bed nucleus of the stria terminalis
CeA: central nucleus of the amygdala
CNS: central nervous system
CRH: corticotropin-releasing hormone
DA: dopamine
DMH: dorsomedial hypothalamic nucleus
DR: dorsal raphe nucleus
EB: embryoid body
ECM: extracellular matrix
EC: endothelial cell
ETV2: ETS variant transcription factor
FGF: fibroblast growth factor
GE: ganglionic eminence
GH: growth hormone
hESCs: human embryonic stem cells
hiPSCs: human induced pluripotent stem cells
hPSCs: human pluripotent stem cells
HPA: hypothalamic-pituitary axis
HRCT: hypocretin
LC: locus caeruleus
LH: luteinizing hormone
MD: major depression
mESCs: mouse embryonic stem cells
ME: median eminence
mPFC: medial prefrontal cortex
NAcc: nucleus accumbens
NCCs: neural crest cells
NE: norepinephrine
NSCs: neural stem cells
OPCs: oligodendrocyte progenitor cells
Pa: paraventricular nucleus of the thalamus
PAG: periaqueductal gray
PD: Parkinson’s disease
PFC: prefrontal cortex
PNS: peripheral nervous system
PRL: prolactin
PVN: paraventricular nucleus of the hypothalamus
RA: retinoic acid
scRNA-seq: single cell RNA sequencing
SHH: sonic hedgehog
SNS: sympathetic nervous system
sgPFC: subgenual prefrontal cortex
TF: transcription factor
VTA: ventral tegmental area.
