# Supplementary material for: From Brain Organoids to Networking Assembloids: Implications for Neuroendocrinology and Stress Medicine
Source: Front Physiol. 2021 Jun 10;12:621970. doi: 10.3389/fphys.2021.621970 (PMC8222922; doi:10.3389/fphys.2021.621970)
Supplement: Supplementary file 1 [file Table_1.docx]

**Table 1. Overview of advances in brain organoid methods to date**.

| **Authors** | **Organoid characteristics** | **Derived from** | **Method** | **Comments** |
| --- | --- | --- | --- | --- |
| Kasai et al., 2020 | Hypothalamic and pituitary (hypothalamic-pituitary unit) | hiPSCs | SFEBq method, FGFs, SAG, BMP4.  (Modified condition):  SAG and BMP4 were started at the same time.  Juxtaposition of hypothalamic neurons and pituitary gland. | Co-existence of hypothalamus and pituitary: ↑↑ ACTH secretion. Regulation of ACTH by hypothalamic CRH and glucocorticoids, as *in vivo*.  Compared with simple induction of the anterior pituitary, this method may reveal more complex relations between tissues.  ACTH-secreting cells function under the control of CRH-secreting cells in a hybrid aggregate. |
| Ozone et al., 2016 | Pituitary | hESCs | SFEBq, SAG, BMP4, FGFs, Notch inhibitors. | Combined application of SHH and BMP4 signals: →hESCs differentiated to oral ectoderm and hypothalamic neurons. Juxtaposition of head and hypothalamic ectoderm. Further exposure to FGF signals induced Rathke’s pouch from ectoderm.  ACTH was regulated by CRH.  Transplantation→  hypophysectomized mice were rescued. |
| Suga et al., 2011 | Pituitary | mESCs | SFEBq,  SAG, BMP4, FGFs,  Notch inhibitors to induce ACTH- producing corticotroph lineage (↓Tbx19, by Notch signaling). | Juxtaposition of head ectoderm and neuroectoderm. Treatment with SHH signaling →multiple endocrine lineages. Focus on corticotrophs to evaluate function. The cells secreted ACTH and responded normally to both positive and negative regulators. Transplantation→  hypophysectomized mice were rescued. |
| Jo et al., 2016 | Midbrain organoids (MOs) | hESCs | Dual SMAD inhibitors, Wnt and SHH pathway activators, FGF8*_._ | Functional dopaminergic and neuromelanin- secreting neurons.TH^+^ neurons showed properties of mature mDAN and expressed functional DA receptors. TH^+^ neurons received inputs from other neurons in the MOs: → generation of functional neuronal networks. |
| Monzel et al., 2017 | MOs | NESCs | Wnt and SHH pathway activators. | Spatially patterned groups of dopaminergic neurons. Mature organoids: Functional synaptic connections with spontaneous activity and functional glial cells (S100B^+^ astrocytes). No microglia or vasculature.  Congruent with brain development *in vivo*: glial differentiation temporally follows neuronal differentiation. |
| Kim et al., 2019 | MOs | hiPSCs | BMP/TGF-β inhibitors, Wnt and SHH pathway activators, FGF8, BDNF, GDNF, AA.  hiPSCs→ dissociated into EBs and 3D organization in Matrigel. | EBs treated with Wnt agonist + BMP/TGF-β inhibitors→neuroectodermal differentiation. FGF8+SAG → mesencephalic fate. Treatment with BDNF, GDNF, AA → differentiation to MOs.  MOs were maintained in BDNF +GDNF, without antioxidant, for 60 days→aging.  PD modeling: Isogenic midbrain organoids. Introduction of LRRK2-G2019S mutation in hiPSCs with CRISPR/Cas9. Transcriptome analysis: ↑↑DA + ↑neuromelanin, resembling aged human midbrain. Add MPTP**→↑ cell death in mutant organoids. PPIN analysis in mutant organoids→ TXNIP: functionally important in pathophysiology of sporadic LRRK2- associated PD. |
| Kwak et al., 2020 | MOs | hESCs | SFEBq, dual SMAD inhibitors, FGF8, SAG, WNT gradient.  Treatment with dual SMAD inhibitors →early neuroectodermal commitment. +Wnt agonist→ regional specification into mesencephalon. +SAG and BMP8→ventralization. MOs embedded into matrigel droplets →basal-apical lamination. | Homogeneous, mature MOs. Structurally and functionally similar to *in vivo* midbrain.  mDANs, neuromelanin, functional synaptic connections and multiple cell types (astrocytes, oligodendrocytes).  Optimal Wnt gradient was needed for establishing the regional identity. Treatment with MPTP**→astrocytes caused selective death of mDANs. Recapitulates *in vivo* pathophysiology mediated by cell-cell interactions and PD phenotype. Suitable for modeling midbrain development and PD. |
| Smits et al., 2019 | Midbrain | mfNPCs | BMP, TGF-β inhibitors,  SHH and WNT signaling activators. | mDANs secreted DA.  Healthy control (H-lines) vs.L-RRK2- G2019S -hiPSCs (P-lines). Mutation → introduced in H-lines, or corrected in P-lines. LRRK2-G2019S MOs: ↓in number + complexity of mDANs compared to control organoids. LRRK2-G2019S- MOs: ↑FOXA2*** in the progenitor cells →neurodevelopmental defect in PD.  Introduction of mutation →effect. Correction of the mutation →no effective rescue. |
| Eura et al., 2020 | Brainstem organoids | hiPSCs, hESCs | FGF2, EGF, BDNF, GDNF, NT-3, cAMP, AA, insulin****, transferrin, progesterone. | Organoids contained midbrain/hindbrain progenitors, noradrenergic, dopaminergic, cholinergic neurons.  IHC: → expression of DBH*****. scRNA-seq analysis + proteomics + electrophysiology: cellular population similar to human brainstem and neural crest→ possible to use hBOs in grafting for transplantation, drug screening, disease modeling. |
| Mugumura et al., 2015 | Cerebellum | hPSCs | SFEBq, FGF2, FGF19, SDF1, insulin. | Induction of isthmic organizer→secondarily self-induces cerebellar tissues. Purkinje cells: morphological and electrophysiological characteristics, similar to *in vivo*. hPSCs self-organized a polarized neuro-epithelial structure in culture. Stepwise addition of patterning factors recapitulated human cerebellar development. FGF19 + SDF1 promoted the self-formation of polarized neural-tube-like structures. Generation of hiPSC-derived Purkinje cells from patients with SCA. |
| Sakaguchi et al., 2015 | Hippocampus | hESCs | SFEBq, BMPs, Wnt agonist (CHIR). | Functional granule- and pyramidal-like neurons from self-organizing telencephalic tissue. Network formation. Steps:  BMP and Wnt signaling →induce choroid plexus (most dorsomedial portion of telencephalon). Titration of BMP and Wnt→self-organization of medial pallium tissues. Recapitulation of hippocampus development.  Hippocampus implicated in several disorders (e.g. AD, schizophrenia). |
| Bagley et al., 2017 | Fused brain organoids (dorsal and ventral forebrain assembloids). | hESCs | SAG, Wnt inhibition.  Use of a single matrigel droplet to embed the organoids. Co-culture. | Organoids grow together and become fused.  Formation of networks. Migrating cells generate multiple MGE-interneuron types (somatostatin, NPY, calbindin, parvalbumin). |
| Birey et al., 2017 | Fused brain organoids (dorsal and ventral forebrain assembloids). | hPSCs | Dual SMAD inhibition, SAG, Wnt inhibition, FGF2, EGF. Static fusion. | Organoids placed adjacent to each other, in a canonical tube. Formation of functional networks. Presence of astrocytes. Interneurons migrated in a saltatory pattern, with nukleokinesis (as *in vivo*). Modeling of Timothy syndrome. |
| Xiang et al, 2017 | Fused human MGE organoids and cortical organoids. | hPSCs | Dual SMAD inhibition, SHH signaling activation, Wnt inhibition.  Static fusion. | Mimicking tangential migration of interneuron progenitors. MGE interneurons. Functional networks. |
| Xiang et al., 2019 | Fused thalamic (TOs) and cortical organoids (COs) | hESCs | Dual SMAD inhibition, insulin, MAPK-ERK blockage →prevents excessive caudalization to midbrain. BMP7→ thalamic differentiation. Further maturation. | Fusion→Thalamo-cortical connections. Modeling reciprocal axon connectivity, in 3D.  Non-cell-autonomous guidance may be required to make efficient axon projections between TOs - COs. Direct contact between TOs-COs may favor axon projections. There might be functional interaction between TOs-COs, involved in maturation of intrinsic properties of thalamic neurons.  Thalamus: relay hub. Thalamic dysfunction implicated in ASD, MD, schizophrenia, epilepsy. Modeling thalamus development, thalamocortical interactions. |
| Madhavan et al., 2018 | Oligocortical spheroids.  Cerebral cortex with myelination. | hPSCs | Pasca et al., 2015 method for human cortical spheroids. PDGF-AA, IGF-1, T3.  PDGF-AA, IGF-1, T3: essential for induction of OPCs and oligodendrocytes. | OPCs and myelinating oligodendrocytes. Week 20: maturing oligodendrocytes and early myelin.  Week 30: myelin compaction. Promyelinating drugs→↑oligodendrocyte generation + myelination. Spheroids recapitulated the pathology of PMD. Induction of all major CNS lineages →new opportunity to study human cortical development + disease→ study demyelination in leukodystrophies or remyelination in MS. Further work needed to refine cortical architecture in spheroids. |
| Marton et al., 2019 | Human oligodendrocyte spheroids (contain neurons, oligodendrocytes, astrocytes, in close proximity). | hiPSCs | Dual SMAD inhibitors, EGF, FGF2, SAG, Wnt inhibitor, PDGF, IGF-1, BDNF, T3, cAMP, NT-3, hepatocyte growth factor, insulin, biotin, AA. | Human oligodendrocyte lineage with multiple stages of oligodendrocyte development, migration, myelination. Neurons, astrocytes, oligodendrocytes: co-developed spatially and temporally→ interactions. Recapitulates oligodendrocyte -neuron, oligodendrocyte –astrocyte interactions. Model similar to *in vivo* development. Further work needed to quantify compact melination→study later stages of oligodendrocyte maturation and myelination. |
| Song et al., 2019a | Brain region-specific organoids (dorsal and ventral) integrated with isogenic microglia-like cells (multilineage assembloids). | hiPSCs | Co-culture of isogenic microglia-like cells with dorsal and ventral cortical spheroids. | Isogenic microglia-like cells showed differential migration ability and immune response. |
| Cederquist et al., 2019 | Forebrain organoids with dorsoventral-and anteroposterior-like polarization (polarized brain organoids). | hPSCs | Steps:  Genetically engineering one hPSC line to express SHH, under the control of doxycycline.  Mixing a small aggregate of cells from this line with a larger aggregate of cells from an unmodified PSC line.  Neural differentiation.  SHH was distributed as a gradient.  Embedding in matrigel (ECM) was not necessary. | Organoids with topographic organization, that became polarized into several topographical forebrain regions: areas resembling LGE or MGE (GSX2^+^or NKX2.1^+^), hypothalamus (NKX2.2^+^ or NKX2.1^+^FOXC1^-^), the thalamus (TCF7L2^+^) and dorsal forebrain (PAX6^+^FOXC1^+^). At later stages, organoids expressed markers for cortical layer-specific neurons (e.g. TBR1^+^), striatal neurons (DARPP32^+^), GABAergic interneurons (LHX6^+^) and hypothalamic neurons (e.g. POMC^+^, OTP^+^).  Limitation: More precise methods to titrate SHH expression are essential. |
| Qian et al., 2020 | Sliced human cortical organoids. | hiPSCs | Slicing method: organoids were “trimmed into a disc shape”, exposing the interior (progenitor zones) to external culture environment. | Disc-shaped organoids received oxygen and nutrients by diffusion through the exposed top and bottom surfaces→↑growth in both directions→ maintaining the organization of cortical structures. Drastically ↓ necrotic area size within organoids (which did not ↑over time) and ↓↓apoptotic cells in the outer regions.  ↑↑size of cortical structures. Model recapitulates late-stage human cortical development: formation of distinct cortical layers.  Caveat: repeated slicing may cause damage to axons and dendrites. |
| Cakir et al., 2019 | Organoids with a functional vascular-like system and BBB characteristics | hESCs | Cortical organoids were infected with ETV2 inducible-containing lentivirus. | Formation of complex vascular-like network →functional maturation of organoids + acquisition of several BBB characteristics: ↑expression of tight junction markers, astrocytic and pericytic proteins and transporters. ↑TEER. ↓hypoxia → ↓interior apoptosis (compared to avascular organoids).  ETV2 induction →functional, permeable, vascularized human cortical organoids.  Potential model to study early prenatal brain, neural–EC interactions in brain development and disease. |
| Bergman et al., 2018 | BBB organoids | human primary brain ECs, astrocytes and pericytes | Brain ECs, pericytes, astrocytes : cultured together in low-adherence condition in 1:1:1 ratio allowed to assemble in multicellular spheroids in 24-48 h. | Expression of features of BBB (tight junctions, molecular transporters, drug efflux pumps). *In vitro* modeling of BBB. |
| Nzou et al., 2018 | Human neurovascular unit organoid model (3D cortex spheroid). | hiPSCs | BMECs, pericytes, astrocytes and neurons: placed into a mixture containing a ratio of 1:1:5:6, respectively. | Spheroids contain BMECs, pericytes, astrocytes, microglia, oligodendrocytes and neurons. ECs enclose the brain parenchymal cells. Expression of tight junctions, adherens junction-associated proteins and cell-specific markers. |
| Pham et al., 2018 | Vascularized whole brain organoids. | hiPSCs | hiPSC-derived whole brain organoids +hiPSCs from the same patient differentiated into ECs. | Matrigel coating of organoids + ECs from the same patient →vascularization of the organoid in 5 weeks. |
| Mansour et al., 2018 | Vascularized human brain organoids. | hESCs | In vivo engraftment model of hPSC-derived brain organoids, in mouse brain. | Grafted organoids with functional vasculature and functional synaptic connectivity. Organoids: progressive neuronal differentiation and maturation, gliogenesis, integration of microglia and growth of axons to multiple regions of the host brain. Functional neuronal networks and blood vessels in the grafts. Graft-to-host functional synaptic connectivity. |
| Song et al., 2019b | Fused neurovascular spheroids  (*multilineage assembloid*) | hiPSC | Fusion of cortical spheroids and isogenic endothelial spheroids, in the presence of supporting MSCs. | Direct mixture of cell types.  The presence of ECs provides BBB-related properties inside COs.↑Notch signaling, matrix remodeling and VEGF-A secretion in assembloids resulted in accelerated cortical tissue development and differentiation. Model for heterotypic cell-cell interactions. |
| Qian et al., 2016; Qian et al., 2018 | Forebrain, midbrain, hypothalamic organoids (region-specific) | hiPSCs | Dual SMAD inhibitors, SHH agonist, selective BMP signaling inhibitor, FGF-2, FGF-8, WNT-3A, CDNF, BDNF, GDNF, AA, cAMP.  SpinΩ (miniaturized multiwell spinning  Bio-reactor). | Addition of patterning factors to EBs→causes differentiation to regional neural progenitor cells (dorsal forebrain, midbrain and hypothalamus).  Then, transfer EBs to SpinΩ: EBs grow and differentiate into organoids, representative of specific brain regions.  Forebrain organoids: embedding EBs in Matrigel→ promotes formation and expansion of large neuroepithelial  structures→ventricular zones.  Balance between intrinsic programming and stochasticity. |
| Pasca et al., 2015 | Human cortical spheroids | hiPSCs | BMP and TGF-β signaling pathways inhibition, FGF2 and EGF. BDNF and NT3. | Mature neurons from both deep and superficial cortical layers: showed spontaneous activity and were surrounded by nonreactive astrocytes. Functional synapses. Network activity. Transcriptionally similar to *in vivo* fetal development. |

AA: ascorbic acid; ACTH: adrenocorticotropic hormone; AD; Alzheimer disease; BBB: blood brain barrier; BDNF: brain-derived neurotrophic factor; BMP: bone morphogenetic protein; BMECs: brain microvascular endothelial cells; CDNF: cerebral dopamine neurotrophic factor; CRH: corticotropin-releasing hormone; COs: cortical organoids; DA: dopamine; DBH: dopamine beta hydroxylase; EBs: embryoid bodies; ECM: extracellular matrix; EC: endothelial cell; EGF: epidermal growth factor; FGF: fibroblast growth factor; GDNF: glial cell line-derived neurotrophic factor; hBOs: human brainstem organoids; hESC: human embryonic stem cell; IGF-1: insulin-like growth factor 1;IHC: immunohistochemistry; ihPSC: induced human pluripotent stem cell; LGE: lateral ganglionic eminence; mfNPCs: midbrain floor plate neural progenitor cells; MAPK-ERK: extracellular signal-regulated kinases pathway; mDAN: midbrain dopaminergic neurons; MGE: medial ganglionic eminence; MOs: midbrain organoids; MSCs: mesenchymal stem cells; MS: multiple sclerosis; MPTP: 2,3,6-tetrahydropyridine; NESCs: human neuroepithelial stem cells; NPY; neuropeptide Y; NT-3: neurotrophin 3; OPCs: oligodendrocyte progenitor cells; PDGF-AA: platelet-derived growth factor; PMD: Pelitzaeus-Merzbacher disease; PD: Parkinson disease; POMC: pro-opiomelanocortin;PPIN: protein-protein interaction network; SAG: smoothened agonist for activation of Sonic hedgehog pathway; scRNA-seq: single-cell RNA-sequence; SCA: spinocerebellar ataxia; SDF1: stroma cell-derived factor; SFEBq: serum-free floating culture of embryoid body-like aggregates with quick reaggregation; SHH: sonic hedgehog; T3: thyroid hormone; TGF-β: transforming growth factor β; TEER: trans-endothelial resistance; TH: tyrosine hydroxylase; TOs: thalamic organoids; TXNIP: Thioredoxin Interacting Protein; VEGF-A: vascular growth endothelial factor A.

*FGF8 is a midbrain-hindbrain boundary derived morphogen;**MPTP: DA neurotoxin; *** FOXA2: floor plate marker required for mDAN generation; ****insulin is a caudalization factor; *****DBH: marker of central noradrenergic neurons.
